# Pharmacokinetics of escalating single‐dose administration of cannabidiol to cats

**DOI:** 10.1111/jvp.13100

**Published:** 2022-10-27

**Authors:** Aaron J. Rozental, Daniel L. Gustafson, Breonna R. Kusick, Lisa R. Bartner, Stephanie Cruz Castro, Stephanie McGrath

**Affiliations:** ^1^ From the Department of Clinical Sciences, College of Veterinary Medicine and Biomedical Sciences Colorado State University Veterinary Fort Collins Colorado USA

**Keywords:** cannabidiol, cannabinoid, cannabis, feline, pharmacokinetics

## Abstract

This study aimed to assess the single‐dose pharmacokinetics and tolerability of a cannabidiol (CBD) isolate in sunflower oil with escalating oral doses in eight healthy, purpose‐bred cats. Eight cats were randomized into six dosing groups of four cats each. Cats were administered a single 2.5, 5, 10, 20, 40, or 80 mg/kg dose orally with at least a two‐week washout in between doses. Behavior scoring, complete blood count, serum biochemistry analysis, physical examination, and CBD plasma levels were evaluated before and after dosing. All cats successfully completed the study. CBD was measured in the plasma of all cats dosed with CBD oil. The Cmax and AUC increased in a dose‐proportional fashion across all dosing groups. There were no major bloodwork or behavioral changes although the BUN and creatinine values decreased after treatment across all doses. No adverse effects were observed, and behavioral changes were limited to head shaking, lip smacking, and hypersalivation immediately following dose administration. Single orally administered CBD doses up to 80 mg/kg were safe and well tolerated in this cohort of cats and display dose‐proportional pharmacokinetics across a broad concentration.

## 1 INTRODUCTION

*Cannabis sativa* is the most well‐known plant containing phytocannabinoids. The two most intensely studied phytocannabinoids from this plant are delta‐9‐tetrahydrocannabidiol (THC) and cannabidiol (CBD). CBD lacks the psychotropic effects of THC and has been a prominent subject of research for medical uses (Brenneisen, 2007). Hemp products (cannabis plant certified to contain less than 0.3% THC by dry weight) have exploded in popularity in recent years largely due to the 2018 Farm Bill (Congress.gov, 2017–2018) which descheduled hemp and hemp seeds and declared hemp an eligible crop under the federal crop insurance program.

Cannabidiol has demonstrated interaction with a wide range of receptors in humans and pre‐clinical models, including CB1, CB2, GPR3, GPR6, GPR 12, GPR55, TRPV1, 5HT1, PPARγ, D2 high, mu, and delta (Peres et al., 2018). There is also evidence that CBD inhibits certain subunits of the cytochrome P450 system (CYP450) of the liver (Yamaori et al., 2011) and may interact with the orexin/hypocretin system (Murillo‐Rodríguez et al., 2019). Given its purported widespread biological activity, CBD has received attention as a potential therapeutic for numerous disease processes.

Cannabidiol has been shown to be well tolerated in dogs (Bartner et al., 2018; Deabold et al., 2019; McGrath et al., 2018; Vaughn et al., 2021) and, in several small studies, has been investigated for use as an anti‐convulsant (McGrath et al., 2019), analgesic (Brioschi et al., 2020; Gamble et al., 2018; Kogan et al., 2020; Verrico et al., 2020), and behavior modifier (Corsetti et al., 2021). Additionally, consumer surveys of cannabis products showed that pet owners are administering hemp products to dogs and cats to treat bacterial and fungal infections, to act as a sleep aid, to treat neoplasia, and to help with digestive tract problems (Kogan, 2018; Kogan et al., 2016). However, no scientific reports have been published as to the usefulness of this phytocannabinoid in cats or whether CBD has the same receptor interaction in this species as other species. Given the recent evidence of differing morphologies, distribution, and functions of cannabinoid receptors across species (Bukiya, 2019), it is important to study CBD's effect in each species, as there may be interspecies differences in tolerability, dose, and effectiveness.

The purpose of this pilot study was to investigate single oral dose pharmacokinetics of CBD in cats in a dose‐escalation study. We hypothesized that a single dose of CBD would not cause severe adverse effects, even at a high dose.

## 2 MATERIALS AND METHODS

This study was carried out under the strict regulations of the Institutional Animal Care and Use Committee (protocol ID: 19‐8448A). As there were no previous studies published at the start of this investigation to guide a power analysis, a power analysis was not performed. Eight healthy adult sexually mature purpose‐bred cats were collected from High‐Quality Research (Fort Collins, CO) and were evaluated (four castrated males and four sexually intact females). There were two calico, two orange tabby, and one each of black tuxedo, brown tabby, grey and white bicolor, and brown and grey tabby cats. The cats were determined to be healthy through physical examinations, CBC, and chemistry panel evaluated by a veterinarian (SM). The animals were given 1 month to acclimate to their new housing and were exclusively fed Iams (Mason, OH) cat food for the duration of their stay. The cats were housed in an on‐site research facility in two separate enclosures (four cats per enclosure). Feeding, cleaning, and evaluation of their overall appearance were performed regularly by Laboratory Animal Resource staff and veterinarians. Additionally, veterinary students provided socialization sessions 2–4 times per week. The cats were an average age of 3.6 years (3.3–3.9 years) and weight of 4.7 kg (3.5–6.3 kg) at the beginning of the trial and an average of 3.8 years (3.5–4.1 years) and 4.9 kg (3.3–6.8 kg) at the end of the trial.

### 2.1 Administration of CBD and sample collection

The CBD product used in this study was composed of a CBD isolate extracted from hemp and a sunflower oil base. Each oil sample was verified as a pure CBD isolate and found to contain CBD within 5% variability from the labeled concentration via third‐party analysis (PhytaTech Denver, CO) commissioned by Canopy Growth Corporation (Smiths Falls, Ontario). The random number generator function in Microsoft Excel (Version 2104) was used to randomly assign the cats regardless of gender into six dosing groups (*n* = 4 cats/group). Each cat received a single oral dose of 2.5, 5, 10, 20, 40, or 80 mg/kg CBD oil followed by at least a two‐week washout period between doses. The eight cats were randomized into six groups of four cats such that each cat was dosed once on each of three different dose administration days. These dose administration days consisted of 2.5 and 5 mg/kg, 10 and 20 mg/kg, and 40 and 80 mg/kg groups such that each cat was evaluated in three different dosing groups with each group being randomized. In an attempt to administer similar volumes of oil between the doses, a 25 mg/ml oil was used for the 2.5 and 5 mg/kg doses, a 100 mg/ml oil was used for the 10 and 20 mg/kg doses and a 200 mg/ml oil was used for the 40 and 80 mg/kg doses. Volumes for each dose can be referenced in Table 1. The oil was administered orally via syringe and was inserted from the side into the mouth past the teeth and squirted onto the caudal aspect of the tongue. This was followed by holding the animal's mouth closed with the head dorsally extended to encourage swallowing.

**TABLE 1:** Total volume of oil administered at each dose (ml).

	Placebo	2.5 mg/kg	5 mg/kg	10 mg/kg	20 mg/kg	40 mg/kg	80 mg/kg
Cat #1	0.60^a^	0.60		0.60		1.10	
Cat #2		0.40		0.45			1.70
Cat #3	1.20	0.65		0.65			2.70
Cat #4		0.35			0.70	0.66	
Cat #5	1.50		1.10		1.20		2.30
Cat #6			1.00	0.50			1.90
Cat #7	1.00		1.00		1.00	1.05	
Cat #8			0.70		0.70	0.68	

*Note*: A table representing the total ml administered to each cat with each dose.

^a^ Error in calculation of this volume.

For jugular vein catheterization, all cats were sedated with 25 μg/kg dexmedetomidine (Zoetis) and 0.5 mg/kg butorphanol (Zoetis) intramuscularly and a 20 gauge catheter was placed in a jugular vein. This process was started in the morning the day before dosing with all jugular catheters in place in the early afternoon. After sedation, each cat's vitals (heart rate, respiratory rate, and temperature) and systolic blood pressure were evaluated via a non‐invasive blood pressure monitor (Doppler). This was performed before dosing to allow for a washout period after sedation before administration of CBD. Baseline CBC and chemistry panels were performed, and additional blood was stored for Time 0 CBD plasma concentration testing. The cats were fasted for at least 8 h and monitored overnight by veterinary students. The next day, each cat was administered the preassigned single oral dose of CBD oil. After dose administration, blood sampling for CBD plasma concentrations occurred at 2, 4, 6, 12, and 24 h for a total of six sample points, including Time 0. Behavior assessments using a behavior scoring system (Quimby et al., 2011) were performed the night before and immediately after dosing and every 60 minutes for the first 6 h. Physical examinations were performed every 2 h for the first 6 h. Additional assessments were performed at 12 and 24 h post‐dosing. The behavior assessments and physical examinations were performed and recorded by whomever was available at the time of the evaluation. As such, scoring was performed by a multitude of individuals including the authors as well as veterinary students. All individuals scored based on descriptions of each score in the scoring system. The jugular catheters were removed 24 h after dosing. If a jugular catheter stopped functioning before the blood draws were completed, the cat was sedated using the same sedation protocol as previously described. Recheck bloodwork (CBC and chemistry panel) was performed 2 weeks after administration of the oil. All cats underwent a minimum of a 2‐week washout period between each new dosing protocol.

All blood samples were collected through the jugular catheter utilizing a three‐syringe technique with a standard heparin‐saline solution. For those patients that required additional sedation due to jugular catheter malfunction, this blood was collected via the saphenous vein. 2 ml of whole blood was put into a sodium heparin blood tube. Each blood sample for CBD plasma levels was centrifuged for 10 min at 2000 *g* and 4°C. The plasma was separated from the red blood cells, placed in 1.5 ml cryotubes, and stored at −80°C until analysis. Samples were processed and frozen within 2 h of collection. Samples that were not processed within 10 min of collection were stored in ice until processing.

### 2.2 Placebo dose administration

Four cats were also included in a placebo dose administration round. They were fasted for 8 h, and e‐collars were placed the night before administration to mimic a normal dosing round. They were not sedated and did not have jugular catheters placed. To calculate the amount of base oil each cat in the placebo group would receive, all prior administered CBD oil volumes were averaged for that individual (Table 1). The next morning, the delivery oil (sunflower oil) was administered to each cat, and behavior scoring and physical examinations were performed as described above for 24 h.

### 2.3 Quantification of CBD in Plasma

Plasma CBD was measured using a validated LC/MS/MS assay by the Pharmacology Laboratory of the Drug Development & Discovery Shared Resource (University of Colorado Cancer Center) (McGrath et al., 2019). Assay performance was monitored using QC samples at three levels (5, 50, and 500 ng/ml) and showed an accuracy and precision (%CV) of 92.5% ± 4.3% across 2 batches with 17/18 QC's passing with greater than 85% accuracy and a lower limit of quantitation of 1.0 ng/ml. The standard curve was established (1–1000 ng/ml) by linear regression with 1/x^2^ weighting.

### 2.4 Statistical analysis

Bloodwork and physical examination analysis: Heart rate was the only physical exam variable that was able to be collected with enough consistency for statistical analysis due to the temperament of the cats (purring at rest, difficult to handle). A mixed model was fit for each response variable separately [heart rate, alkaline phosphatase (ALP), alanine aminotransferase (ALT), blood urea nitrogen (BUN), creatinine, basophils, eosinophils, neutrophils, and mean corpuscular volume (MCV)]. Specifically, dose, time, and dose*time interaction were included as fixed effects. Cat and cat*dose were included as random effects to account for the repeated measures design. For each time point, comparisons were made between doses with a Tukey adjustment. For each dose, comparisons were made for downstream time points vs Time 0. For heart rate, a Dunnett adjustment was used. Model assumptions were evaluated by visual inspection of residual diagnostic plots. ALT (log), basophils (square root), and neutrophils (log) were used to better satisfy model assumptions. *p* values < .05 were considered significant.

Behavior analysis: As the behavior score variables were not normally distributed, non‐parametric approaches were used for analysis. Prior to analysis, the initial behavior scores 24 h before and at the time of dose administration were averaged and the time points between 1–12 h after dosing were also averaged. Comparisons were prepared between doses using the Kruskal‐Wallis test. A Bonferroni adjustment was performed separately for each behavior to account for multiple time points. For each dose, comparisons were prepared for downstream time points vs baseline (Time 0) using the Wilcoxon signed‐rank test.

Pharmacokinetic parameters were calculated by non‐compartmental analysis using Phoenix 64 WinNonLin v.8.3.3.33 (Certara). Parameters analyzed included maximal concentration (Cmax), time to Cmax (Tmax), area under the curve (AUC_0‐24_), half‐life (T_1/2_), mean resonance time (MRT), apparent clearance (Cl/F), and apparent volume of distribution (Vd/F) for CBD. The number of points for calculating the terminal half‐life was three. Cmax and Tmax were determined directly from the data. Dose proportionality of AUC was determined using a one‐sample t‐test and Wilcoxon test. Dose proportionality of Cmax was determined via a linear regression analysis.

## 3 RESULTS

All of the CBD concentrations in the oil were within 5% variability of the labeled dose. A CBD isolate was used, and the oil was produced from a single batch. The labelled 25, 100, and 200 mg/ml oils were analyzed to contain 26.18, 103.83, and 207.98 mg/ml of CBD, respectively. An error in calculation resulted in the incorrect volume of placebo oil administered to one cat (Table 1).

### 3.1 Pharmacokinetic results

**FIGURE 1:**
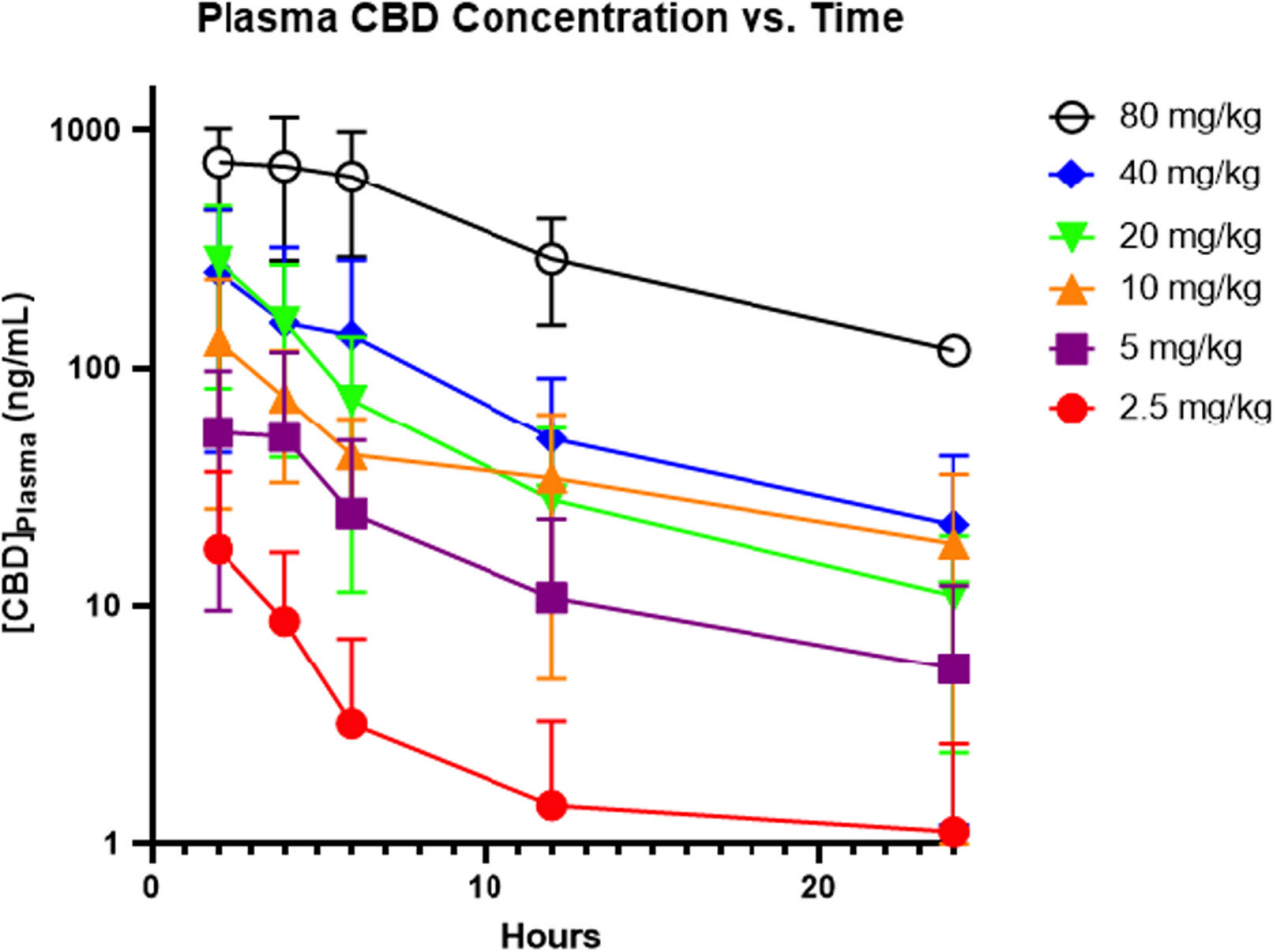
Median CBD plasma concentration with standard deviation bars with respect to time after a single oral administration of CBD (2.5, 5, 10, 20, 40, and 80 mg/kg) to cats.

**FIGURE 2:**
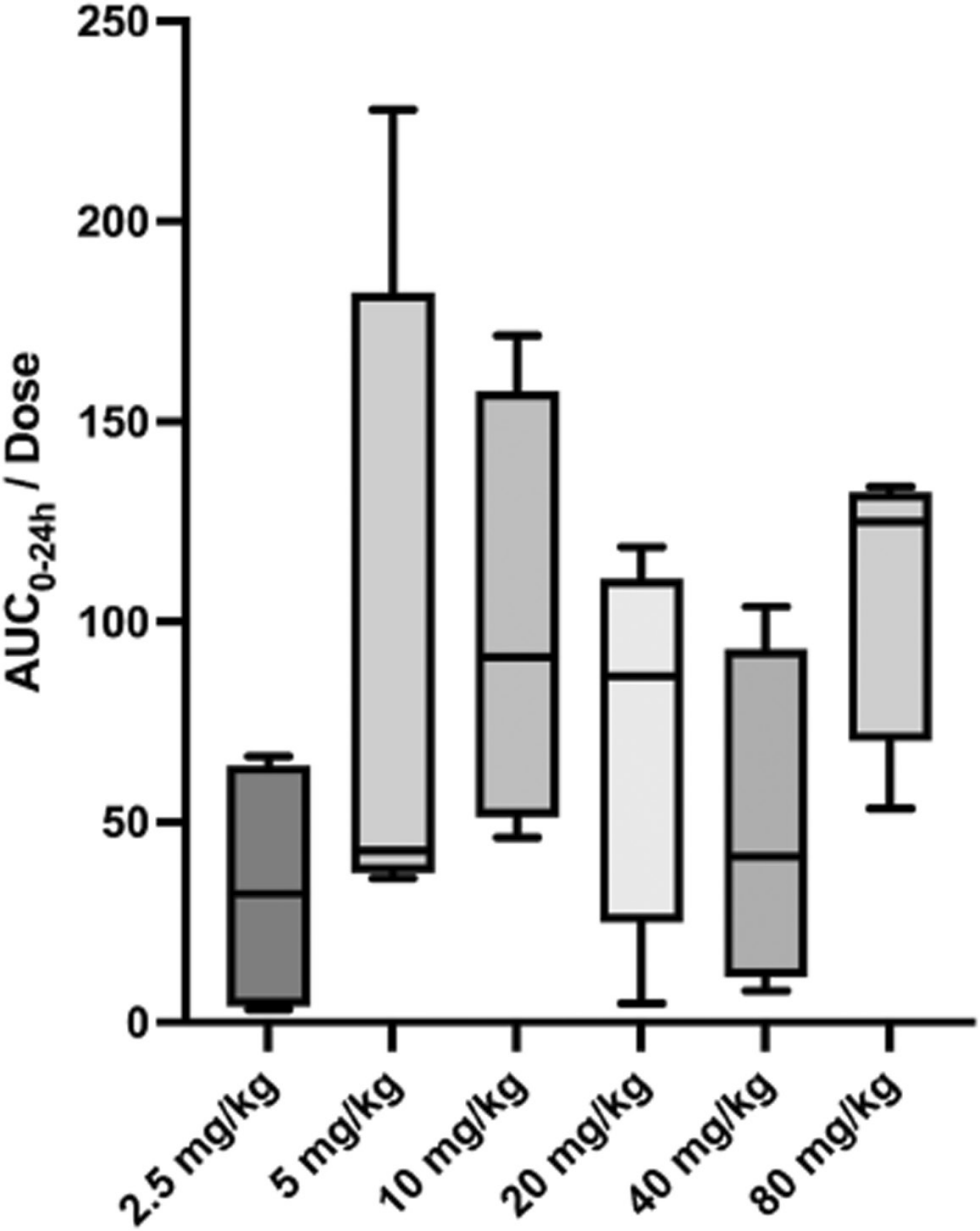
Box and whisker plot depicting AUC_0‐24h_/dose for each dosing group. AUC_0‐24_ was determined to be dose‐proportional using a one‐sample *t*‐test and Wilcoxon test.

All eight cats successfully finished the study. Each of the CBD doses (2.5, 5, 10, 20, 40, and 80 mg/kg) was orally administered (*n* = 4 cats/dosing group). Plasma CBD concentrations were measured at six timepoints (0, 2, 4, 6, 12, and 24 h). A single cat in the 10 mg/kg dosing group required sedation for its 12 and 24‐h blood draws due to dislodgement of the jugular catheter. A summary of the means of Cmax, AUC_0__‐24_, T_1__/2_, MRT, clearance, Vd/F, and median Tmax results can be seen in Table 2 and Figure 1. The mean T_1__/2_ ranged from 6.7 to 13.2 h across all groups. However, the T_1__/2_ was unable to be calculated for two cats in the 2.5 mg/kg and 80 mg/kg group as well as for one cat in the 10 mg/kg group. This was also the case for these same groups when calculating the clearance and Vd/F due to not having measurable concentration in two cats in those dosing groups. A linear regression analysis was performed plotting Cmax values vs. dose, which showed a linear relationship with the slope significantly different from zero (*r* ^2^ = .9235; *p* value = .0023). This suggests that Cmax was proportional to dose. A one‐sample *t*‐test and Wilcoxon test showed that AUC_0__‐24_/dose was not statistically significantly different, suggesting AUC_0__‐24_ also increased in a dose‐proportional fashion (Figure 2). The mean AUC_0__‐24_ was 8738.1 (4269.6–10690.0) ng/ml × h in the 80 mg/kg dosing group. The median Tmax was 2.0–3.0 h in all dosing groups. The mean clearance ranged from 7.13–37.3 L/h/kg across all dosing groups. There were two cats in the 80 mg/kg group that had measurable CBD plasma levels at Time 0, which in this case was following a four‐month washout period due to a delay in the study related to the COVID‐19 global pandemic.

**TABLE 2:** Pharmacokinetic parameters of CBD across all doses.

Parameter	Dose (mg/kg)					
	2.5	5	10	20	40	80
C_max_ (ng/ml)	17.8 (3.2–45.3)	61.1 (19.9–148.5)	132.6 (43.2–258.4)	281.0 (14.5–467.4)	251.7 (47.4–467.0)	963.9 (744.6–1126.8)
T_max_ (h)	2.0 (2.0–4.0)	2.0 (2.0–4.0)	2.0 (2.0–24.0)	2.0 (2.0–4.0)	2.0 (2.0)	3.0 (2.0–6.0)
Half‐Life (h)	13.2^a^ (12.8–13.5)	8.2 (6.0–11.0)	7.5^b^ (6.2–8.5)	9.0 (5.7–14.9)	9.6 (6.3–17.7)	6.7^a^ (6.6–6.7)
AUC_0‐24_ (ng/ml × h)	83.5 (8.1–165.9)	437.1 (180.0–1139.2)	1000.4 (460.8–1714.1)	1481.0 (92.9–2372.9)	1945.8 (313.1–4150.1)	8738.1 (4269.6–10690.0)
CL/F (L/h/kg)	12.6^a^ (12.3–13.0)	17.4 (3.7–23.1)	11.1^b^ (5.3–20.2)	45.2 (8.5–84.6)	37.3 (7.7–71.8)	7.13^a^ (6.8–7.5)
MRT (h)	4.5 (2.2–8.5)	7.0 (5.9–8.1)	9.0 (7.0–13.9)	6.3 (7.2–8.1)	7.5 (8.1–15.1)	8.4 (7.6–9.4)
Vd/F (L/kg)	239.7^a^ (239.6–239.8)	196.9 (50.2–344.7)	110.1^b^ (65.1–179.1)	886.6 (73.1–3246.3)	702.0 (89.0–2152.5)	68.3^a^ (65.8–70.9)

*Note*: A table listing pharmacokinetic parameters (C_max_, T_max_, Half‐Life, AUC_0‐inf_, CL/F, MRT, and Vd/F) for all doses. Values represent the mean value for four animals except where noted, and the range of the values is shown below in parentheses. Median Tmax is represented.

^a^ PK parameters could only be calculated for two of the four animals treated at this dose.

^b^ PK parameters could only be calculated for three of the four animals treated at this dose.

### 3.2 CBC and serum biochemistry results

There was a statistically significant correlation between creatinine and BUN concentrations at Time 0 and Time 24. Both creatinine and BUN consistently decreased between Time 0 and Time 24 measurements by an average of 0.23 and 5.7 mg/dl respectively which was statistically significant (Table 3). Creatinine was consistently higher than 1.6 mg/dl pre‐dosing – which is above the reference interval for the diagnosis of CKD based on IRIS staging (International Renal Interest Society, 2019). There were otherwise no statistically significant changes to any other CBC or serum biochemistry values between Time 0 and the Time 24 evaluations. Creatine kinase activity was also consistently elevated at the 24‐h blood draw compared to Time 0.

**TABLE 3:** Time 0 and time 24 BUN and creatinine across all doses

	Mean Creatinine Concentration (mg/dl)		Mean BUN Concentration (mg/dl)	
	Time 0	Time 24	Time 0	Time 24
2.5 mg/kg	1.6 ± 0.2	1.3 ± 0.2^a^	27.8 ± 3.4	22.8 ± 3.3^a^
5 mg/kg	1.7 ± 0.4	1.3 ± 0.3^a^	24.3 ± 1.3	17.0 ± 1.4^a^
10 mg/kg	1.7 ± 0.2	1.5 ± 0.1^a^	25.5 ± 2.4	21.3 ± 3.4^a^
20 mg/kg	1.6 ± 0.3	1.3 ± 0.2^a^	25.5 ± 3.7	19.0 ± 5.0^a^
40 mg/kg	1.3 ± 0.2	1.3 ± 0.3	24.0 ± 1.4	20.5 ± 4.0^a^
80 mg/kg	1.6 ± 0.3	1.4 ± 0.3^a^	28.0 ± 2.2	20.3 ± 3.0^a^

*Note*: A table presenting mean serum creatinine and BUN concentrations at time 0 and time 24 at each dose. The International Renal Interest Society considers 1.5 mg/dl to be the upper limit for normal creatinine concentration in cats. The normal reference value for BUN at the laboratory used in this study was 18–35 mg/dl. Values represent the mean creatinine and BUN values for all cats with standard deviation at time 0 and time 24. Statistical significance is denoted with an asterisk at time 24 when compared to time 0.

^a^ *p* value < .05 for significance.

### 3.3 Behavior

There were no statistically significant changes in behavior scoring (activity, interaction, and vocalization) after CBD administration or placebo at any dose.

### 3.4 Adverse events

No adverse events were noted. Behavioral changes including head shaking, lip smacking, and hypersalivation immediately after dose administration were noted consistently. Blood pressure was able to be collected after sedation in 19 of 24 attempts. Using previously established systolic values for normotension (Acierno et al., 2018; Payne et al., 2017), one cat was classified as hypotensive after sedation (87 mmHg) and three cats were classified as hypertensive (220, 180, and 158 mmHg) under sedation. There were otherwise no observed adverse events associated with CBD dosing.

## 4 DISCUSSION

This pharmacokinetic study evaluated escalating single doses of oral CBD oil in healthy cats. While there are multiple proposed methods of CBD administration (inhalation, gel capsule, oil/tincture, and topical), we chose to dose the cats in this study with CBD oil orally based on the previous pharmacokinetic work in dogs suggesting this may be one of the more reliable methods of CBD delivery (Bartner et al., 2018). Cats (*n* = 4 cats/group) received 2.5, 5, 10, 20, 40, and 80 mg/kg doses. The CBD plasma pharmacokinetics were measured over 24 h. In this study, we were able to successfully demonstrate the CBD exposure and safety of a single administration of oral doses up to 80 mg/kg of highly purified CBD oil to a small cohort of healthy cats.

Overall, there was a large amount of inter‐individual variability across all doses and parameters (Table 2). For instance, the AUC after administration of a 2.5 mg/kg dose ranged from 8.1–165.9 ng/ml × h. This is about a 20‐fold difference between these individual cats. We suspect that as a lipid‐soluble drug, the fat content of each individual may serve as a reservoir for its uptake (Routledge, 1985). Given the large differences in body condition that we see across this population of cats, this may significantly affect its interindividual pharmacokinetic properties. This highlights the necessity of future studies to continue to evaluate the pharmacokinetics in a larger cohort of cats as well as to evaluate CBD plasma levels in treatment studies.

Although there was a large inter‐individual variability, the mean Cmax and AUC_0‐24_ increased proportional to the dose.

There was some variability in the mean T_1/2_ between the 2.5 mg/kg dosing group and the rest of the groups with a longer T_1/2_ (13.2 h) in the 2.5 mg/kg dosing group._._ This variation may be related to the fact that this value was calculated from two cats and may not represent the true mean. Excluding the 2.5 mg/kg dose, the mean T_1/2_ ranged from 6.7–9.6 h across all other dosing groups.

There were no statistically significant changes to any of the parameters in the CBC in this study. When evaluating serum biochemistry analysis, there was a trend towards decreasing BUN and creatinine at 24 h when compared to Time 0 (Table 3). This was consistent and statistically significant among all dosing groups with p values consistently below 0.05. Approximately 42% of the Time 0 creatinine values were ≥1.6 μg/dl, which is the normal cutoff for chronic kidney disease in cats by the International Renal Interest Society (International Renal Interest Society, 2019). We do not have an explanation for this or why these values consistently decreased at the 24‐h blood draw. There was only a single cat that developed hypotension under sedation while all other values were within the normal limits or hypertensive. So, it would seem that hypotension or hypertension while under sedation was unlikely to be the cause. The significance of decreasing creatinine and BUN values 24 h after dose administration is unknown. This must be evaluated in future studies to investigate the repeatability of this finding. In many cats, the creatine kinase showed increased activity 24 h after CBD administration. This was attributed to jugular catheter placement. There was no elevation in ALT or ALP post‐dose in any dosing groups as has been seen in chronic administration in humans, dogs, and in a single cat (Bartner et al., 2018; Deabold et al., 2019; Scheffer et al., 2021; Vaughn et al., 2021).

There has been one previous report describing single‐dose CBD pharmacokinetics and safety in cats (n = 6) (Deabold et al., 2019). In this previous report, hypersalivation was noted after dosing. A 1:1 mixture of CBD and CBDA was delivered in fish oil capsules at a 2 mg/kg dose, the equivalent of 1 mg/kg CBD. The Tmax was 2.0 h, AUC was 164 ± 29 ng/ml × h, and Cmax was 43 ng/ml ± 9. A larger dose (2.5 mg/kg) in our study yielded a median Tmax of 2.0 h. The mean Cmax and AUC_0‐24_ were considerably lower (17.8 ng/ml and 83.5 ng/ml × h, respectively). However, the high end of the Cmax range in our study was 45.3 ng/ml which is comparable to the previous study. The discrepancies between these investigations could be due to differences in the delivery method (oil capsule versus liquid oil), a consequence of the differences in the make‐up of the CBD oil itself, the substantial interindividual variability observed with oral CBD administration, or the small sample sizes in the studies. A small human study suggested that THCA‐A and CBDA enhance the bioavailability of the non‐acidic forms of THC and CBD (Eichler et al., 2012). As the previous feline pharmacokinetic study used a combination of CBD and CBDA in their oil, this could account for difference in bioavailability. However, additional research is needed to understand the influence of CBDA on CBD bioavailability in cats.

Another study in felines evaluated escalating doses of a CBD predominant oil, in which the Cmax and Tmax of a 25 mg/kg dose was evaluated following nine previously escalating doses each 3 days apart (Kulpa et al., 2021). Cmax concentration was measured to be 236.0 ± 193.0 ng/ml at this 25 mg/kg dose with a mean Tmax of 3 h. This is comparable to the current study in which a mean Cmax of 321.0 (14.5–467.4) ng/ml was achieved in the 20 mg/kg dosing group with a median Tmax of 2.0 h. However, it is not clear that 3 days between dosing provides an adequate washout period. As such, the CBD plasma concentrations likely did not start at zero which makes a full comparison to the current study difficult. This investigation also evaluated a THC/CBD combination oil. Following escalating doses with pharmacokinetics performed after a 34 mg/kg THC and 10.6 mg/kg CBD, a CBD Cmax was achieved almost double that of CBD oil alone. This suggests that THC may enhance the absorption of CBD. As such, both of these previous studies may indicate that the combination of CBD with other phytocannabinoids may enhance absorption of CBD.

There were no observed adverse events in the course of this study. In addition, there were no statistically significant changes to each cat's behavior when analyzed with a numerical behavior assessment. However, behavioral changes included lip licking, head shaking, and hypersalivation immediately after administration of the oil in the treatment and placebo groups. Other adverse events including, emesis, diarrhea, somnolence, muscle tremors, mydriasis, pruritis, ataxia, and hypothermia have been reported previously in canine and feline patients but were not observed in this study (Bartner et al., 2018; Chicoine et al., 2020; Deabold et al., 2019; Fernández‐Trapero et al., 2020; Gamble et al., 2018; Vaughn et al., 2020, 2021). However, some of the aforementioned studies included THC in their formulations as well as chronic administration which may affect pharmacokinetics and physiological effects. It is interesting to note that previous studies showed no notable differences in adverse event frequency between doses of CBD oil and delivery oil alone (placebo) administered to dogs although a statistical analysis was not evaluated to compare the adverse event profile (Vaughn et al., 2020, 2021). This finding was not mirrored in a similar feline study and instead suggested a greater occurrence of hypersalivation and lethargy in the treatment group as compared with the MCT oil placebo group (Kulpa et al., 2021). This same study also found a statistically significant difference in adverse events between the administration of MCT oil alone and sunflower oil alone suggesting fewer adverse events with the administration of sunflower oil. Most of the adverse events with MCT oil were vomiting or diarrhea, while gastro‐intestinal adverse events with sunflower oil were minimal (*n* = 1) (Kulpa et al., 2021).

Whether there are sex‐dependent differences in CBD pharmacokinetics remains to be seen. We were unable to determine with certainty in this cohort of cats whether there are sex‐dependent differences in the pharmacokinetics of CBD due to the small number of participants. Future studies to evaluate for differences in pharmacokinetics in males vs. females and intact or castrated animals should be performed.

All cats were fasted in this study as we were unable to standardize meals for these free‐fed cats. Given previous research in dogs, humans, and pre‐clinical models showing that THC and CBD oils administered in fed or fasted states changes the bioavailability of THC (Birnbaum et al., 2019; Łebkowska‐Wieruszewska et al., 2019; Millar et al., 2018; Zgair et al., 2016), future studies could evaluate the pharmacokinetics of CBD in fasted and post‐prandial states in cats. Along the same lines, one might suspect that varying volumes of CBD oil in the fasted state may alter bioavailability of the CBD. However, in this study we were able to show dose proportionality across all doses for AUC_0‐24_. This suggests that although the volumes of CBD oil administered varied with varying doses (Table 1), the bioavailability was constant so the volume of oil was not a confounder in this study.

Limitations of this study include the use of a non‐validated behavior scoring system, though it has been used in a previous study (Quimby et al., 2011). Although clear definitions exist for each score, multiple evaluators may have led to varying results. Additionally, the small sample size may not allow detection of subtle changes to behavior. A single cat in the 10 mg/kg dosing group required sedation for its 12 and 24‐h blood draws due to dislodgement of the jugular catheter. It is unlikely that this affected the pharmacokinetics of the CBD oil at these time points, but may have affected behavior scores. However, it is unlikely that these data points would have altered the statistical significance of the behavior scores in this study. In addition, given the initial sampling for CBD levels every 2 h, it is possible we may have missed the true Cmax and Tmax. This may affect the reported Cmax in this study as the true Cmax may have been larger. Every 2‐h sampling may also have led to AUC being underestimated, which would have led to overestimation of CL/F and Vd/F. More frequent sampling would have allowed for more accurate information regarding absorption. Additional sampling after 24 h may have allowed for better characterization of the terminal phase as well.

There were two cats in the 80 mg/kg group that had measurable CBD plasma levels at Time 0 (<70 ng/ml). These cats had had a four‐month washout from their previous dosing which was a 10 mg/kg dose. One explanation could be storage of CBD in fat tissues as is seen with THC in humans (Gunasekaran et al., 2009), but this may not necessarily explain why the prolonged washout period was not suitable for this group. However, all other cats had no measurable plasma CBD at Time 0. More data are needed with regard to interindividual variability relating to clearance and half‐life.

Overall, we have found that single doses of CBD oil up to 80 mg/kg were well tolerated in this cohort of cats. However, safety and pharmacokinetic studies evaluating chronic CBD dosing are needed. In addition, this pilot study now provides statistical variables to aid in designing larger controlled pharmacokinetic studies. The half‐life would indicate that once to twice daily dosing would be appropriate for future studies to assess the therapeutic potential of CBD in cats. However, CBD efficacy for use as a medication and required plasma concentrations for these effects have yet to be evaluated.

## AUTHOR CONTRIBUTIONS

All authors contributed substantially to the manuscript in the following manner: (1) Have prepared substantial contributions to conception and design, or acquisition of data, or analysis and interpretation of data; (2) Been involved in drafting the manuscript or revising it critically for important intellectual content; (3) Given final approval of the version to be published; (4) Agreed to be accountable for all aspects of the work in ensuring that questions related to the accuracy or integrity of any part of the work are appropriately investigated and resolved.

## CONFLICT OF INTEREST

Stephanie McGrath was a paid consultant of Canopy Growth Corporation.

## ANIMAL WELFARE AND ETHICS STATEMENT

Animals were cared for under the strict regulations of the Institutional Animal Care and Use Committee (protocol ID: 19‐8448A).

## Data Availability

Data are available upon reasonable request from the author.
